# 伴微乳头结构肺腺癌的研究进展

**DOI:** 10.3779/j.issn.1009-3419.2015.11.08

**Published:** 2015-11-20

**Authors:** 祥宇 石, 青松 庞, 纲 赵, 路军 赵, 平 王

**Affiliations:** 1 300060 天津，天津医科大学肿瘤医院放疗科 Department of Radiotherapy, Tianjin Medical University Cancer Institute and Hospital, National Clinical Research Center of Cancer, Key Laboratory of Cancer Prevention and Therapy, Tianjin 300060, China; 2 300060 天津，天津医科大学肿瘤医院病理科 Department of Pathology, Tianjin Medical University Cancer Institute and Hospital, National Clinical Research Center of Cancer, Key Laboratory of Cancer Prevention and Therapy, Tianjin 300060, China

**Keywords:** 肺肿瘤, 治疗, 预后, Lung neoplasms, Therapeutics, Prognosis

## Abstract

伴微乳头结构(micropapillary pattern, MPP)肺腺癌是一种临床少见的具有高度侵袭性的恶性肿瘤，近年来因其高死亡率被人们所重视。2011年关于肺腺癌的病理新分类将其作为一种独立的病理类型，此后针对该类肺癌个体化治疗的相关研究逐渐展开。近期的相关研究发现，伴MPP肺腺癌在转移机制、临床病理学、影像学、治疗及预后方面具有显著异质性。本文对伴MPP肺腺癌转移机制及相关临床研究进展进行探讨。

## Abstract (en)

Lung adenocarcinoma with micropapillary pattern (MPP) is a kind of rare high invasive malignant tumor, which has been noticed because of high mortality. In 2011, the new pathological classification of lung adenocarcinoma classify it as an independent pathological type, researches on the individual treatment of the disease had been gradually expanded. Recent studies have demonstrated that lung adenocarcinoma with MPP has obvious heterogeneities in metastasis mechanism, clinical pathology, imageology, therapeusis and prognosis. In this paper, we discuss the progress of metastasis mechanism and clinical relevance in lung adenocarcinoma with MPP.

非小细胞肺癌(non-small cell lung cancer, NSCLC)是目前世界上发病率和死亡率最高的恶性肿瘤之一，肺腺癌作为NSCLC中最常见的组织学类型，近年来的发病率在多数国家呈上升趋势，其在临床、病理学和分子学等方面的异质性被广泛研究。

微乳头结构(micropapillary pattern, MPP)是指游离的中央缺乏纤维血管的细胞簇。伴有MPP结构的癌在过去的二十年中逐渐被人们认识，Amin^[1]^于2002年首次报道伴有MPP的肺腺癌，并认为其更容易发生远处转移。世界卫生组织(World Health Organization, WHO)肺癌组织学分类(2004版)简单描述了伴MPP肺腺癌，之后国内外学者对其开展了大量研究，逐渐认识到MPP分子生物学行为独特，伴MPP肺腺癌具有侵袭性强，淋巴结转移率高，易早期远处转移等多种预后不良特征^[1-4]^。因此，2011年国际肺癌研究协会(International Association for the Study of Lung Cancer, IASLC)/美国胸科协会(American Thoracic Society, ATS)/欧洲呼吸协会(European Respiratory Society, ERS)关于肺腺癌分类建议中将含MPP＞5%递增量的浸润性肺腺癌单独划分为一个新的组织学类型：微乳头为主型腺癌(micorpapillary predominant adenocarcinoma, MPA)。本文对伴MPP肺腺癌在治疗、预后及其他方面的进展予以综述。

## 1 MPP转移机制

上皮细胞-间叶细胞转化(epithelial to mesenchymal transition, EMT)在肿瘤发生转移过程中扮演重要角色^[5]^。近期研究发现伴MPP肺腺癌易早期发生淋巴结转移的特征或与此相关。EMT过程中，肿瘤上皮细胞间E-钙粘蛋白介导的细胞间粘附消失，逐渐发生向间质细胞的转变，同时间叶细胞特异性标志物波形蛋白表达增高。Kamiya^[4]^发现含有MPP成分的肿瘤细胞间E-钙粘蛋白表达降低，另外，两项研究^[6, 7]^均发现含MPP肿瘤细胞高表达波形蛋白。

## 2 临床病理学

肺腺癌中，MPP常与其他组织类型混合存在，在肿瘤组织中所占比例从1%-90%不等，目前还未发现100%MPP亚型^[8]^。与其他类型肺腺癌细胞相比，多项研究^[9-12]^发现伴MPP肺腺癌细胞体积较大，胞质嗜酸性，细颗粒状，核质比较高，胞核呈中等异型性，部分病例中可见沙砾体结构^[2, 13]^。统计显示，MPP在男性^[9, 11, 14]^及非吸烟人群^[14, 15]^中更易出现。

## 3 影像学

含MPP肺腺癌侵袭度高，病情进展迅速，通过术前影像学检查对MPP进行早期识别尤其重要。Cha等^[9]^通过计算机断层扫描(computed tomography, CT)和正电子发射型计算机断层显像(positron emission computed tomography, PET)-CT分析了511例肺腺癌患者，发现临床分期＞Ⅰ期，肿瘤大小≥2.5 cm，无磨玻璃样结构的实体肿块和标准化摄取值(standard uptake value, SUV)最大值≥7是存在MPP的独立预测因素，且当该四项因素同时满足时准确率可达100%。对于早期浸润性肺腺癌，有研究^[16]^发现不同病理类型之间SUV值存在差别，其中MPA患者SUV值明显增高。虽然目前关于通过影像学早期诊断MPP的研究尚少，但利用功能影像及代谢生物标记技术对MPP组织学亚型进行术前探测是可行的。

## 4 治疗

### 4.1 手术

肺叶切除术加纵隔及肺门淋巴结清扫术是早期肺癌手术治疗的金标准^[17, 18]^。但对于病灶较小(≤2 cm)的早期肺腺癌，很多研究发现局部切除术与根治性切除术有效率相同，同时还可保留肺功能^[19, 20]^。因此肿瘤大小(≤2 cm)是目前早期肺癌术式选择的唯一标准^[21, 22]^。在小病灶(≤2 cm)肺腺癌中，MPP的重要性日益受到关注，MPP≥5%的小病灶(≤2 cm)肺腺癌患者局部切除术后复发风险增高^[8, 23]^。早期小病灶(≤2 cm)MPA患者或许应行较大范围的肺叶切除术以降低局部复发风险。

### 4.2 术后辅助治疗

对于Ⅰb期浸润性肺腺癌，单纯手术治疗的MPA患者中位总生存期(overall survival, OS)及中位无病生存期(disease free survival, DFS)均低于其他类型^[24]^，因此是否可通过增加术后辅助治疗以提高生存值得讨论。有研究^[25]^发现包含MPA在内的高度恶性浸润性肺腺癌对铂类为基础的化疗敏感性高，术后辅助化疗可提高MPA患者DFS^[26]^，但Hung^[10]^的研究未发现辅助化疗能够提高包含MPA患者在内的中位OS及中位疾病特异性生存期，Warth^[27]^更是发现接受铂类为基础的辅助化疗的MPA患者中位OS及DFS均低于未行化疗患者。目前的研究尚未发现辅助化疗可明显改善MPA患者OS，还需要前瞻性研究及相关基础研究的进一步探索。

Warth等^[27]^研究发现放疗未能提高Ⅲ期/Ⅳ期术后浸润性肺腺癌患者的生存。已知术后放疗可显著延长N2期NSCLC患者生存期^[28, 29]^，N2期MPA患者术后放疗的效果仍然未知。关于MPA患者能否通过放射治疗生存获益仍需大样本数据的验证及相关实验分析来探索。

### 4.3 分子靶向治疗

#### 4.3.1 表皮生长因子受体(epidermal growth factor receptor, EGFR)

*EGFR*基因突变常见于患有腺癌、无吸烟史的亚裔女性患者。近期研究^[30-35]^发现，伴MPP肺腺癌*EGFR*基因突变高于其他类型。Motoi等^[36]^最先发现伴MPP肺腺癌患者的*EGFR*基因突变率增高。随后，依据国际肺癌研究协会(The International Association for the Study of Lung Cancer, IASLC)/美国胸科学会(American Thoracic Society, ATS)/欧洲呼吸学会(European Respiratory Society, ERS)MPA关于肺腺癌分类建议分类的相关研究均支持这一结论^[34, 37, 38]^。因此MPP或许可作为存在*EGFR*基因突变的一项重要预测因素。EGFR酪氨酸激酶抑制剂(tyrosine kinase inhibitor, TKIs)已成为*EGFR*基因突变的NSCLC患者一线用药^[39]^，但对于存在*EGFR*突变的伴MPP肺腺癌，EGFR-TKIs的作用仍然未知。Sumiyoshi^[11]^的研究中，6例术后复发的伴MPP腺癌患者同时存在*EGFR*基因突变，其中5例使用EGFR-TKIs后获得长期生存。Zhang等^[32]^研究了21例MPA，其中20例存在*EGFR*基因突变，随访发现EGFR-TKIs可降低该类患者复发率。然而，还需要更多的临床研究探索*EGFR*突变的伴MPP肺腺癌患者能否通过EGFR-TKIs获益。由以上研究可以看出，伴MPP肺腺癌患者*EGFR*突变率高，且小样本数据显示EGFR-TKI治疗有效，在传统治疗对该类疾病疗效较差的基础上，EGFR-TKI或许能成为该类患者的首选治疗。

#### 4.3.2 间变性淋巴瘤激酶(anaplastic lymphoma kinase, ALK)

*ALK*基因是肺癌诱发基因之一，Nishino等^[40]^研究发现MPP与*ALK*基因突变具有相关性，约22%伴MPP肺腺癌患者*ALK*基因发生了突变。同时有研究^[41]^认为MPP与ALK阳性率不具有相关性。目前已知，*ALK*基因阳性率与*EGFR*、*KRAS*基因突变率呈负相关，NSCLC患者中ALK表达和*EGFR*、*KRAS*突变很少同时存在，而MPP是*EGFR*突变影响因素之一。这或许可以解释为什么伴MPP患者*ALK*基因阳性率低。

#### 4.3.3 c-Met酪氨酸激酶(c-Met)

*c*-*Met*基因在肿瘤细胞的转移及侵袭中起重要作用。Koga等^[42]^发现磷酸化c-Met水平高表达与MPP阳性肿瘤(MPP≥10%)及淋巴结转移相关。且伴有磷酸化c-Met高表达的Ⅰa期患者预后低于低表达者(5年OS：51.3% *vs* 79.4%)。另外，MPP阳性组中，磷酸化c-Met高表达与淋巴结转移相关。这一发现将c-Met活化同MPP形态的发展及肿瘤侵袭淋巴系统的生物学机理联系起来。

#### 4.3.4 鼠类肉瘤滤过性毒菌致癌同源体B1(BRAF)

*BRAF*基因是人类最重要的原癌基因之一，关于该基因在肺癌中的研究刚刚起步，*BRAF*基因在NSCLC中的突变率约为0.7%-3%^[43-45]^。De Oliveira Duarte Achcar^[46]^最早分析了伴MPP的肺腺癌与*BRAF*突变的关系，并在15例患者中发现3例存在*BRAF*突变。Marchetti^[47]^的研究发现，发生*BRAF*突变肺腺癌患者中，80%存在MPP。然而近期几项研究均未发现MPA与*BRAF*突变有关^[32, 34, 37, 48]^。

#### 4.3.5 *RB*基因通路

Choi等^[49]^对早期肺腺癌*RB*基因通路突变与预后相关性进行研究时发现，包含MPA及实性为主型在内的高侵袭性肺腺癌的突变基因具有显著多样性。此结论或许能为肺腺癌的个体化靶向治疗提供新的突变位点。

## 5 预后

Miyoshi等^[13]^发现含MPP可影响早期肺腺癌患者的预后，MPP所占比例越大，预后越差。Hirano^[6]^的研究得到同样的结论。2011年IASLC/ATS/ERS关于肺腺癌分类认为MPP与预后不良呈明显正相关，建议将其单独归类为一项病理类型^[50]^。随后来自全球各地的一系列研究对这一划分类型进行验证，多数学者^[3, 10, 12, 15, 27, 33, 35, 51-55]^发现新分类在细胞结构、临床病理特征及预后各方面均有明显分层，并认为含MPP的肿瘤具有高侵袭性，即使对其早期行根治性切除依然难以避免高复发率和相对较差的预后。另外，Lee等^[56]^的研究发现，即使浸润性肺腺癌MPP＜5%，其仍然是降低OS的影响因素。对于进展期浸润性肺腺癌，Campos-Parra等^[25]^对257例Ⅲb期-Ⅳ期的浸润性肺腺癌患者的资料进行分析后得出了相反的结论，他们发现包含MPA在内的高度恶性组的中位OS及中位无进展生存期(progression-free survival, PFS)均高于鳞屑样结构为主型及腺泡样结构为主型组成的低度恶性组。但是该研究对组织学亚型进行评估时采用的是小组织切片而非手术后切除标本对实验结论的准确性有一定影响。

## 6 结论

EMT可能是引起伴MPP肺腺癌预后差、易早期侵犯淋巴结的一项重要因素。对于原发灶较小(≤2 cm)的早期肺腺癌，伴MPP提示局部复发风险高，术前应采取措施尽早探查MPP，利用功能影像及代谢生物标记技术探测这一结构是可行的。目前放疗及化疗对伴MPP的肺腺癌并未取得明显疗效，但结论仍需更多大样本数据验证。靶向治疗方面，伴MPP肺腺癌*EGFR*突变率高，但目前EGFR-TKIs对该类患者疗效仍然未知。关于其他几种靶点基因在伴MPP的腺癌中突变的研究仍处于起步阶段，尚未形成定论。
